# Sustainable planning: the case study of the Strait of Messina ports

**DOI:** 10.1007/s11356-023-31764-2

**Published:** 2024-01-31

**Authors:** Daniele Enea, Alberto Mastrilli

**Affiliations:** Italian National Agency for New Technologies, Energy and Sustainable Economic Development (ENEA), 90133 Palermo, Italy

**Keywords:** Energy and environmental planning, Energy efficiency, Port authority, Photovoltaic plants, LNG, Cold ironing, Strait of Messina

## Abstract

The energy and environmental policy carried out by the Port System Authority of the Strait (AdSP), in charge of the management of the ports spread along the Strait of Messina, is reported. The Environmental and Energy Planning Document of Port Systems (DEASP, the Italian acronym) is the document explaining the AdSP sustainable strategy to reduce GHG emissions. It defines specific measures, in order to improve energy efficiency in buildings and infrastructures, promote the use of renewable energy in the port area, and confer environmental benefits for the citizens of neighboring territories and port users. The main actions developed are as follows: photovoltaic solar plants and tidal energy systems, electrification of the docks to allow the shore supply of ships, and the construction of a Liquefied Natural Gas (LNG) storage plant to replace more polluting marine fuels, together with awareness campaigns on “green” issues, involving the 3 million users of these ports. Starting from the socio-economic and environmental analysis of the territorial context managed by AdSP, the DEASP analyses all the activities carried out inside port areas and reports the energy consumptions of the concessionaries, in the way to calculate the carbon footprint and develop an environmental sustainable strategy to reduce pollutant emissions. The interventions foreseen are assessed through the cost-benefit analysis and allow reducing the GHG emissions in 2030 up to 46%.

## Introduction

**Fig. 1:**
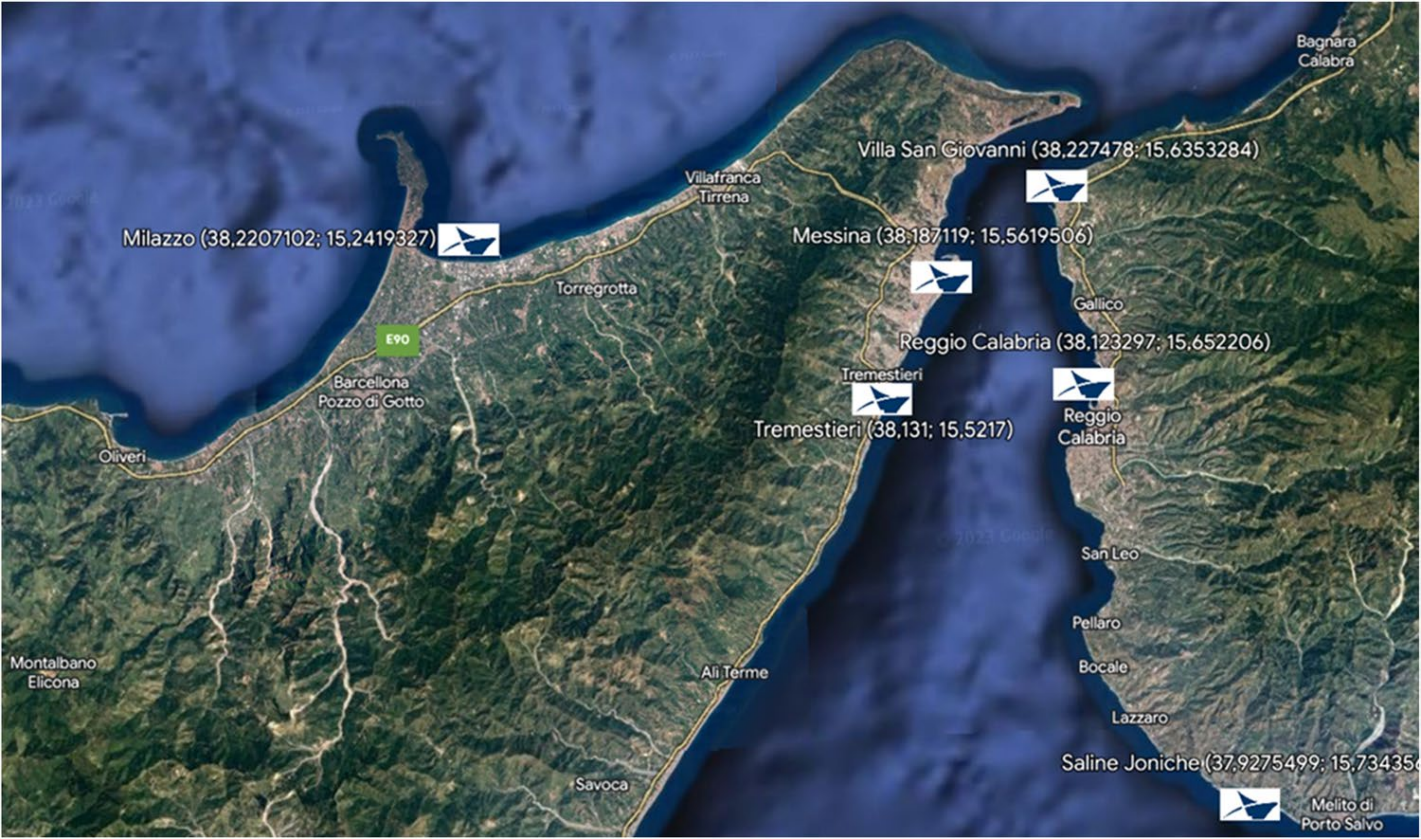
The ports under the administrative control of AdSP. On the left the Sicilian ports, on the right the Calabrian ones

The regulatory regime, within which the planning of port areas is framed, was deeply innovated in Italy by Legislative Decree 4 August 2016, n.169. This legislative decree was provided with the reorganization, rationalization, and simplification of the discipline concerning the port authorities, according to the previous L. 28 January 1994, n. 84 and L. 7 August 2015, n. 124 (Ferrari and Merk 2015; Loffreda and Settani 2021; Luise et al. 2021). The management and control of the Italian ports were divided into 15 port system authorities, and the ports spread along the Strait of Messina were under the government of the Port Authority of the Southern Tyrrhenian and Ionian seas and the Strait of Messina, including also the ports of Gioia Tauro, Crotone, Corigliano Calabro, Taureana di Palmi, and Vibo Valentia. Only later, the L. 17 December 2018, n. 136 introduced a modification, renaming the port authority and reducing the number of ports under its control, thus instituting the Port System Authority of the Strait (AdSP). AdSP is the legal entity endowed with financial autonomy, in charge of the direction, programming, control, coordination, promotion of port operations, and other commercial and industrial activities carried out in the ports of Messina, Tremestieri and Milazzo, in Sicily, and Villa San Giovanni, Reggio Calabria and Saline Joniche, in Calabria (Fig. 1).

Due to their strategic role to manage very high-energy microcosms, more and more the role of port authorities is evolving becoming proactive to promote energy efficiency in all the activities, the adoption of new technologies, among which particularly the use of renewable energy sources (Acciaro et al. 2014; Wiegmans and Geerlings 2010).

Port authorities are growingly point on the development of the “Green port” concept, an integrated system to manage and support marine environment protection and modernization of facilities in seaports (Badurina et al. 2017). The Three-year Operational Plan (TOP) is one of the Port Authority System’s main strategic planning documents outlining the priority interventions and the development strategies for port and logistic activities in the ports of competence (Aveta and Romano 2020). The drafting of the Environmental Energy Planning Document of System Port Authority (DEASP), another fundamental strategic environmental and energy plan, was introduced in Italy by the Legislative Decree 169/2016, and it has to be drafted in accordance with the guidelines published by the Ministry of the Environment and the Protection of the Territory and the Sea (MATTM). The principle of energy sustainability has to be as a permeating element of the planning of the port system, in line with the policies promoted by the current European directives and with the regional and national planning tools, with a specific focus on the reduction of carbon dioxide emissions.

## The DOCKS project

In order to comply with the law and produce the DEASP, AdSP decided to participate to the 1^st^ European New Energy Solutions Optimized for islands (NESOI) call to obtain financial and technical support, both in form of direct funding and advisory services, provided by the NESOI consortium (NESOI 2020). AdSP thus submitted the Development Of Consistent Key strategy of the Strait port system (DOCKS) proposal and resulted beneficiary of the NESOI support, starting the activities in September 2021. Together with SINLOC and RINA, part of the NESOI consortium, AdSP involved the Mediterranean University of Reggio Calabria, as a coordinator, ENEA, and the CNR-ITAE of Messina, in the drafting of the DEASP, for the ports under the administrative management of AdSP. The DEASP defines strategic guidelines for the implementation of specific measures, towards the clean energy transition of the ports, in order to improve energy efficiency and promote the use of renewable energy, thus providing environmental benefits for the citizens of the neighboring territories and for the port users and was finally approved by AdSP in the late September 2022 (Mega et al. 2022). The planning of the port system was developed respecting the criteria of energy and environmental sustainability, in accordance with European and Italian policies with a priority objective: the reduction of CO_2_ emissions. In this way, the objective of energy and environmental planning was the respect of the 33% reduction of CO_2_ emissions in 2030, compared to 2005, imposed by the Italian Integrated National Energy and Climate Plan (PNIEC). The mapping of regional, national, and European planning tools was defined in order to ensure the consistency of the planning actions with the current framework, particularly the three-year operational plan (POT) 2020–2022, approved on 07/08/2020, and the port strategic plans. According to the MATTM guidelines, the DEASP was organized into 5 items: the actual state of the AdSP ports, the regulatory framework, the carbon footprint, the energy and environmental strategy, and the interventions’ program.

### The actual state of the AdSP ports

The socio-economic, territorial and environmental analysis were firstly carried out, in terms of infrastructures, passenger and freight traffic, territorial and environmental context: land and marine morphology, landscape and environmental restrictions, biodiversity and seismic risk. This analysis showed the penalization of Sicily and Calabria, if compared to all other European regions, in terms of higher transport costs (Persyn et al. 2019). All the ports of Messina and Milazzo, in Sicily, and Reggio Calabria and Villa San Giovanni, in Calabria, were deeply described and analyzed, collecting the last available data, regarding with port operator activities, lighting systems and ship, road and railway traffic, thanks to the support of the port concessionaires and AdSP. The ports of Tremestieri and Saline Joniche were not included in the analysis due to very limited presence of concessionaries and port activities. The main data in terms of concessionaries and state-owned areas are listed in Table 1, showing Milazzo has the wider state-owned areas, mainly due to the 640,000 m^2^ area, managed by the co-operative joint-stock company refinery of Milazzo, and the 90,000 m^2^ area, managed by the local thermoelectric power plant.

**Table 1:** Main AdSP ports general data, 2021

	Messina	Milazzo	Reggio Calabria (RC)	Villa San Giovanni (VSG)
No. of concessionaries	57	32	28	3
State-owned areas (m^2^)	425,815	792,757	48,068	19,939

According to data on port goods, listed in Table 2, Milazzo is the only port among those controlled by AdSP, used for liquid bulk trade, particularly crude oil, gaseous, liquefied and compressed petroleum products, and natural gas. In the port of Milazzo, there is, also, wide minerals, cements and limes trade, corresponding to the AdSP 80% solid bulk trade, the rest is settled in the port of Reggio Calabria. Packaged goods are traded in all the AdSP ports, mainly in the ports of Messina (50.5%) and Villa San Giovanni (41.6%). Regarding goods trade, the pandemic emergency, with the lockdown from March to May 2020, produced a decrease in all fields in 2020, that was regained in 2021. In 2022, the more significant data refer to liquid bulk that in the port of Milazzo almost recovers the value of pre-pandemic period (−5% respect 2019). Furthermore, the contraction of the solid bulk in the port of Milazzo is evident (−66% respect 2019), where traffic was concentrated exclusively on steel products, has been affected by the Russian-Ukrainian conflict (AdSP 2023).

**Table 2:** Main AdSP port goods’ data (2019−2022)

		Messina	Milazzo	RC	VSG
2019	Lb	0	17,856,829	0	0
	Sb	0	154,808	42,921	0
	Pg	6,119,264	259,738	688,706	5,039,904
2020	Lb	0	14,880,732	0	0
	Sb	0	162,732	40,550	0
	Pg	5,775,281	212,990	669,927	4,729,989
2021	Lb	0	15,206,560	0	0
	Sb	0	220,212	53,400	0
	Pg	7,131,391	220,212	705,271	6,024,348
2022	Lb	0	17,017,423	0	0
	Sb	0	53,248	57,854	0
	Pg	6,554,607	274,553	709,459	5,435,880

*Lb* liquid bulk [1,000 t], *Sb* solid bulk [1,000 t], *Pg* packaged goods [1,000 t]

Data on regular and cruise passengers are reported in Table 3. These data show the high impact of pandemic on passengers traffic, with a 38% mean decrease of regular traffic in 2020, that is partially regained in 2021. The 2020 partial complete closure of passengers traffic, also, produced 95.4% decrease in cruise traffic across the port of Messina, the only port of AdSP able to the reception of cruise ships. The 2021 data show the recovery of cruise traffic, even if not at the same level of 2019. All data concerning regular passengers show an increase of over 20% for each port of AdSP, while cruise passengers in the port of Messina show a significant increase (+148%) respect 2021 and not yet at the level of the pre-pandemic period (−11%).

**Table 3:** Main AdSP passengers’ data (2019–2022)

		Messina	Milazzo	RC	VSG
2019	Rp	10,755,431	1,116,763	793,045	9,875,455
	Cp	422,732	0	0	0
2020	Rp	6,547,508	697,442	480,512	6,013,867
	ΔRp	−39.1	−37.5	−39.4	−39.1
	Cp	19,537	0	0	0
	ΔCp	−95.4	0	0	0
2021	Rp	7,769,231	846,241	531,859	7,180,910
	ΔRp	+18.8	+22.2	+11.2	+19.6
	Cp	156,322	0	0	0
	ΔCp	+87.5	0	0	0
2022	Rp	9,562,749	1,020,385	649,266	8,847,511
	ΔRp	+23.1	+20.6	+22.1	+23.2
	Cp	387,632	0	0	0
	ΔCp	+148.0	0	0	0

*RP* regular passengers, *Cp* cruise passengers, Δ*Rp* percentage regular passengers variation respect the previous year [%], Δ*Cp* percentage cruise passengers variation respect the previous year [%]

### Energy consumption of the port system

All port concessionaries were involved in the consultation phase by the distribution of an energy questionnaire by e-mail, thanks to the administrative support of AdSP, collecting the records of all concessionaries. No. 36 of them participated to the study, sending the information required. The analysis of the concessionaries showed No. 60 of them do not have fuel and electricity consumptions, thus the response rate was equal to 76%. The consumptions of the other concessionaries were estimated, based on occupied surface area. Data were collected between September and November 2021, per port site (Milazzo, Messina, Tremestieri, Villa San Giovanni and Reggio Calabria), regarding with the two-year period (2019–2020), in terms of

- Electricity purchased from the national grid (considering a yield of 0.42)
- Self-produced electricity on site, by renewable energy plants
- Fuels (natural gas, diesel, petrol, LPG, etc.) used in stationary combustion systems (boilers, generators or cogeneration plants)
- Fuels (natural gas, diesel, petrol, LPG, etc.) used in mobile combustion systems (ships, goods handling machines, cranes, trucks, cars, etc.)

In particular, energy consumptions within the port sites were attributed to the following:

- Concessionaries (electricity)
- Concessionaries (fuels)
- Public lighting
- Ship traffic
- Road traffic
- Railway traffic

**Fig. 2:**
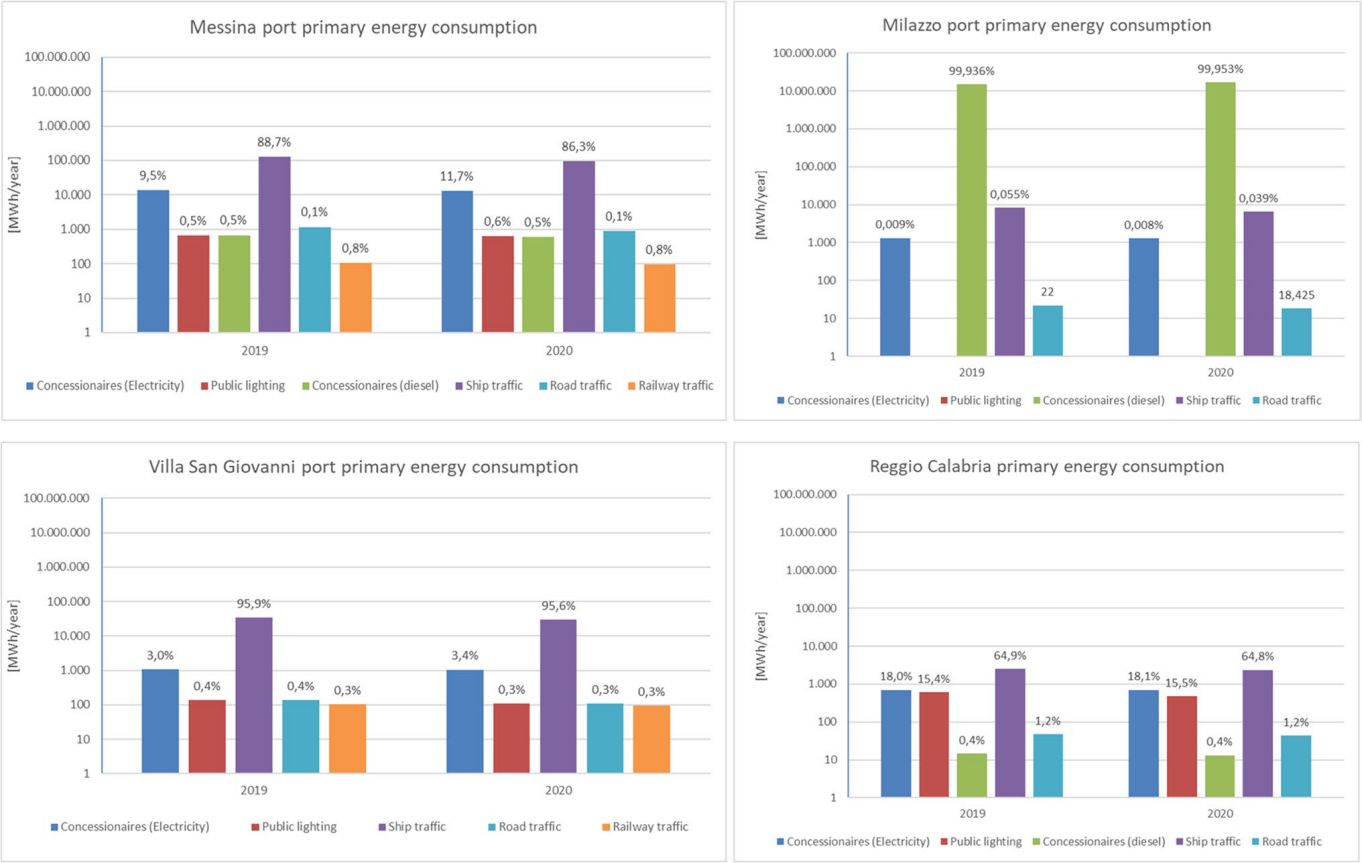
Primary energy consumption of the AdSP ports, per source typology

Particularly, data per hour on mooring in the port and maneuvering and transported cargo (tons of goods and number of passengers and vehicles transported including railway carriages) for each vessel with stopovers in the ports of AdSP and data on energy consumption (electricity purchased from the national grid, self-produced electricity on site from renewable source plants, fossil fuels used in stationary, and mobile combustion systems) were collected thanks to the support of the AdSP concessionaries, as shown in Fig. 2.

Data shows the majority of primary energy consumption in the port of Milazzo, up to 99.15% of the total consumption in the AdSP ports, due to the presence of a refinery and a power plant, as concessionaires of AdSP, with 1.55 million MWh and 15.26 million MWh, in 2020, mostly depending on their diesel consumption. The distribution per source is reported in Table 4, thus showing the low contribution of railway traffic, road traffic and public lighting, respect with the other sources; ship traffic was responsible of 170,000 MWh in 2019, undergoing 21% decrease in 2020, due to the COVID 19 lockdown, as even shown in Table 3. The total consumption was also assessed not considering the two energy intensive industrial plants (EI) set partly in the state-owned area of the Milazzo port. The same 21% decrease between 2019 and 2020 was reported considering all the concessionaries without the EI, with a total amount of 150,771 MWh in 2020.

**Table 4:** AdSP ports’ total primary energy consumption [MWh]

Source	2019 (including EI)	2019 (excluding EI)	2020 (including EI)	2020 (excluding EI)
Concessionaries (electricity)	16,524	15,974	15,853	14,545
Public lighting	1412	1412	1214	1214
Concessionaires (diesel and gas)	14,875,549	689	16,812,775	612
Ship traffic	170,583	170,583	133,158	133,158
Road traffic	1366	1366	1051	1051
Railway traffic	206	206	191	191
**Total consumption**	**15,065,640**	**190,230**	**16,964,241**	**150,771**

### Greenhouse gas evaluation

The greenhouse gas emissions including carbon dioxide (CO_2_), methane (CH_4_), and nitrous oxide (N_2_O), expressed in CO_2e_ from port systems, particularly from ships inside the ports, were deeply studied. Before presenting the DEASP results, we report some statistical data referring to global level of GHG emissions. According to the 4th Greenhouse Gas Study 2020 (IMO 2021), the GHG emissions of total shipping (international, domestic and fishing) have increased from 977 million tons in 2012 to 1076 million tons in 2018 (9.6% increase). In 2012, 962 million tons were CO_2_ emissions, while in 2018 this amount grew 9.3% to 1056 million tons of CO_2_ emissions. The share of shipping emissions in global anthropogenic emissions has increased from 2.76% in 2012 to 2.89% in 2018. Styhre et al. (2017) reported on four several case-studies in Sweden, Australia, Japan, and the USA, showing the contribution of GHG emissions quantified in the ports of Gothenburg, Long Beach, Osaka, and Sydney, using a model developed by the IVL Swedish Environmental Research Institute and proposing four emission-reduction measures and proposing several measures to reduce emissions in the ports. The methodology used for estimating emissions from port operators’ activities and ship, road, and railway traffic, per single port and overall, is based on the indications given in the guidelines for the drafting of the DEASP and on the methodology of the EMEP/EEA air pollutant emission inventory guidebook 2019. GHG emissions were therefore evaluated starting from the energy vectors’ consumption in the three-year period, 2018–2020, even taking into account any self-production/self-consumption. The emissions due to naval traffic were calculated considering the power of the vessels and the times required for the phases of landing, stationing, departure, and the emissions from road traffic according to the distance traveled in the port area for each category of vehicles. The GHG emissions due to railway traffic, only for the ports of Messina and Villa San Giovanni, were calculated based on the distance traveled by railway wagons from the station to the ferryboats, whose operations take place using diesel-shunting locomotives. For each source listed above, GHG emissions were calculated according to the recommended methodology.

#### Emissions from port operators’ activities

GHG emissions were calculated as follows:

$${E}_j^k={C}_j{FE}_j^k$$

where *C* [MWh] is the total energy consumption, *FE* [t/MWh] is the emission factor of the indicator *k*, used to evaluate the Carbon Footprint (CO_2_, CO_2eq_, LCA), and *j* is the energy vector. The indicators used to estimate the Carbon Footprint are the following:

- Emissions of CO_2_ only
- CO_2_ equivalent emissions, with the contribution of other greenhouse gases evaluated with the *GWP* conversion factor, taken from the 2006 IPCC Guidelines (IPCC 2006) with a time horizon of 100 years
- LCA emissions of CO_2_ equivalent, which take into account the entire life cycle of the energy vector

The emission factors used for the calculation of CO_2_ are reported in the literature. In the cases in which we proceeded starting from the emissions of individual greenhouse gas, the result of the emissions in terms of CO_2_ equivalent was obtained as follows:

$${E}_j^{\textrm{C}{\textrm{O}}_{2,\textrm{eq}}}=\sum\nolimits_{k=1}^N GW{P}^k\times {E}_j^k\ \left[{\textrm{tCO}}_{2\textrm{eq}}\right]$$

Where *GWP* is the Global Warming Potential, *E* [t] is the pollutant emission, *j* is the energy vector, and *k* is the greenhouse gas. The GHG taken into consideration were CO_2_, CH_4_, and N_2_O, having GWP for several time horizons (20, 50, 100 years). Emissions were assessed on the basis of consumption declared by the concessionaries, when available, or on the basis of average values estimated for similar activities.

#### Emissions from naval traffic

The assessment of polluting emissions from naval traffic needed the calculation of energy consumption due to ships in the port area, produced during landing and departure maneuvers and during the stationary phase. The calculation was conducted with the 2019 EMEP/EEA methodology relating to naval traffic. The energy consumption of a single type of vessel is calculated as follows:

$${CE}_{n,k}={P}_n{t}_{n,k}\left({L}_{n,k, MAIN}\ F{T}_k+{R}_{A/M}\ {L}_{n,k, AUX}\right)\ \left[\textrm{kWh}/\textrm{phase}\right]$$

where *P* is the maximum engine power [kW], *L* is the load factor, calculated as the ratio between the power engaged in the single phase with respect to the maximum power of the engine, *FT*_*k*_ is the percentage of operational time of the main engine [%], *R*_*A/M*_ is the ratio between the power of the auxiliary engine and the power of the main engine (1 for the main engine), *t* is the time [h], *n* is the type of vessel, *k* is the phase (shunting, stationing), and *MAIN* refers to the main engine and *AUX* to the auxiliary engine. The total energy consumption in the reference period is calculated as follows:

$${CE}_j={10}^{-3}{N}_j\sum\nolimits_{n=1}^{N_n}\sum\nolimits_{k=1}^3C{E}_{n,k}\ \left[\textrm{MWh}\right]$$

where *N* is the number of vessels in the period, *j* is the type of fuel, *k* is the phase, varying from 1 to 3 (departure, landing, stationing), *n* is the type of vessel, and *N*_*n*_ is the number of ship types. The total emissions were calculated as follows:

$${E}^k=\sum\nolimits_{j=1}^{N_c}{CE}_j{FE}_j^k\ \left[\textrm{t}\right]$$

where *CE* is the total energy consumption in the port area, *FE* [t/MWh] is the emission factor of the indicators *j* referring to fuel and *k* used to evaluate the carbon footprint (CO_2_, CO_2eq_, and LCA), and *N*_*c*_ is the number of fuels.

#### Emissions from road traffic

The calculation of emissions due to road traffic was conducted with reference to the approach of the methodology reported in the 2019 EMEP/EEA guidelines; the emissions for each pollutant gas in the period were calculated using the following equation:

$${E}_v^k={10}^{-6}{N}_v{d}_v{FE}_v^k\ \left[\textrm{t}\right]$$

where *N*_*v*_ is the total number of vehicles of category *v*, *d*_*v*_ [km] is the average distance traveled in the port area by category *v* vehicles, and *FE* [g/km] is the emission factor of the pollutant *k* for category *v *vehicles. The pollutant emission factors were obtained from the 2019 database of average emission factors of road transport in Italy of the Istituto Superiore per la Protezione e la Ricerca Ambientale (ISPRA). The emission factors per driving cycle in urban areas referring to the year 2019 were used (ISPRA 2019).

#### Emissions from railway traffic

Polluting emissions due to railway traffic are due to the movement of railway wagons from the railway station to the ferry boats. The operations take place using diesel shunting locomotives. The emissions, for each pollutant taken into consideration, were determined as follows:

$${E}^k= CC\times F{E}^k\ \left[\textrm{t}\right]$$

where *CC* [t] is the total fuel consumption in the port area, *FE* [kg/t] is the emission factor of the pollutant, and *k* is the pollutant (CO_2_, CH_4_, and N_2_O). The emissions in terms of CO_2_ equivalent were calculated as follows:

$${E}^{C{O}_{2, eq}}=\sum\nolimits_{k=1}^N GW{P}^k\times {E}^k\ \left[\textrm{t}\right]$$

where *GWP* is the Global Warming Potential, *E* [t] is the pollutant emission and *k* the pollutant (CO_2_, CH_4_, and N_2_O).

#### Total GHG emissions

**Fig. 3:**
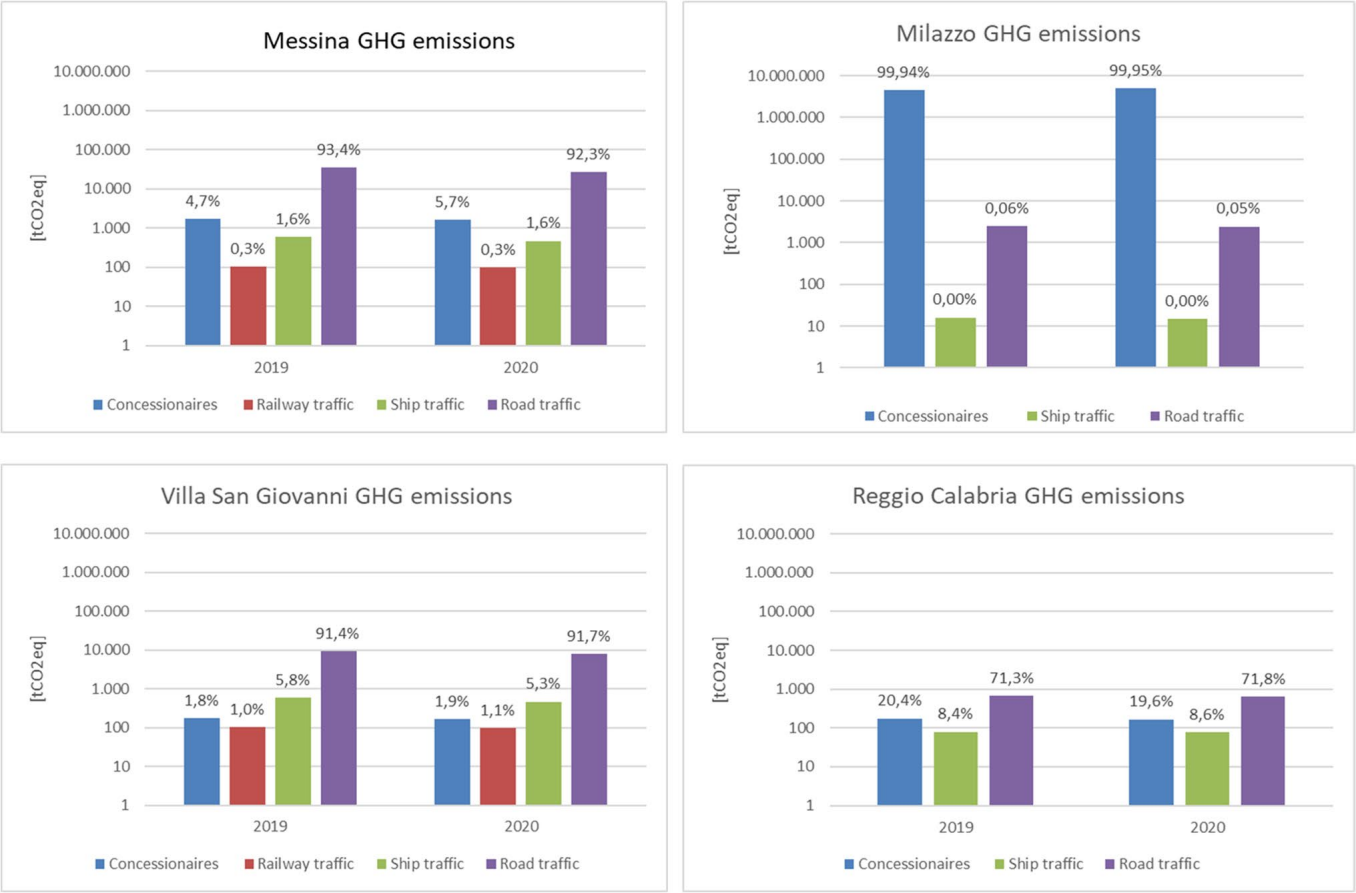
GHG emissions of the AdSP ports, per source typology

The total GHG emissions calculated per port are reported in Fig. 3, using the logarithmic scale, to better represent the high differences, per different source.

This analysis showed GHG emissions up to 4.5 million tCO_2equ_ in 2019 and 5 million tCO_2equ_ in 2020, mostly located in the port of Milazzo, due to the presence of the refinery and the power plant. Table 5 shows the total GHG emissions per source and reflecting the total primary energy consumption, the higher level of GHG emissions are produced by concessionaries, followed by ship traffic. Road and railway traffic produce low GHG emissions. The total amount of GHG emissions was also calculated not considering the two energy intensive concessionaries, set in Milazzo, as the interventions AdSP intends to realize do not directly involve these two industrial plants. The evaluation of GHG emissions data in Table 5 leads to highlight the main weight of the two EI on the total amount, being up to two order of magnitude respect the other sources of emissions. Without considering the EI, the most relevant source of GHG emissions in the port context is ship traffic.

**Table 5:** AdSP ports’ total GHG emissions [tCO_2equ_]

Source	2019 (including EI)	2019 (excluding EI)	2020 (including EI)	2020 (excluding EI)
Concessionaries	4,477,784	2175	5,035,738	2045
Ship traffic	46,476	46,476	37,521	37,521
Road traffic	1254	1254	1022	1022
Railway traffic	208	208	192	192
**Total GHG emissions**	**4,525,722**	**50,113**	**5,074,473**	**40,780**

### The environmental and energy strategy

The environmental and energy strategy were planned by AdSP concerns of 3 main pillars:

1. Reduction of GHG emissions from boats and vehicles
2. Reduction of energy consumption of buildings and port infrastructures
3. Renewable energy sources (RES)

**Fig. 4:**
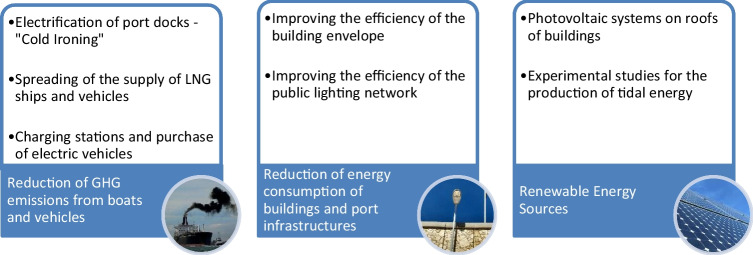
The energy and environmental strategy of the DEASP related to the main interventions

These pillars point to the creation of *green ports*, by identifying the potential opportunities for energy transition and decarbonization. The most promising interventions are the following: cold ironing, aimed at supplying electricity to ships during their mooring in port, the potential conversion of ships to new fuels as LNG and hydrogen, energy production from renewable sources (solar and tidal), energy efficiency of port assets (public lighting, buildings), and electric mobility (Fig. 4). Two of the proposed measures are coherent with the results of the study by Styhre et al. (2017): on-shore power supply and the use of alternative fuels, together with reduced turnaround time at berth and reduced speed in fairway channels.

### The interventions derived from the energy and environmental strategy

Before introducing the main interventions occurring to realize the DEASP environmental and energy strategy, we report the interventions programmed by the two EI. The refinery of Milazzo presented No. 3 projects:

- The production of green methanol, starting from green hydrogen and CO_2_
- The production of biofuel from vegetable oils used and from biomass
- The production of bioethanol from agri-food processing waste

The total amount of investment is 22.7 M€ to reduce environmental impact (Raffineria di Milazzo 2021).

The A2A thermoelectric power plant in Milazzo intends to transform the industrial site into a new integrated energy pole. Several projects will be realized:

- Construction of a treatment and recovery plant for the organic fraction of municipal waste with 75,000 ton/y treatment capacity, producing bio-methane and certified quality compost as fertilizer in agriculture
- Plastic treatment plant with 50,000 ton/y capacity
- 3 MW photovoltaic plant
- 850 MW combined cycle gas turbine plant (CCGT)

The total amount of investment will be 450 M€ in the next 10 years (A2A 2021).

#### Cold ironing

**Fig. 5:**
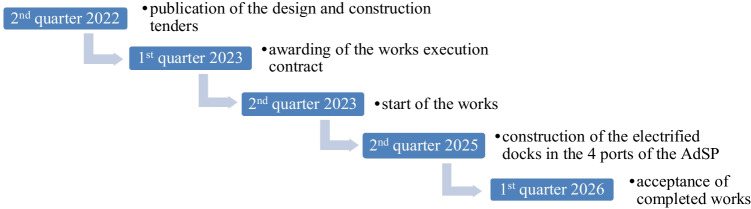
Time schedule of the Stretto Verde action

Cold ironing means the construction of electrical infrastructures in the docks, specially designed to power moored ships; it confers the environmental benefit to reduce CO_2_, NOx, PM_10_, and PM_2,5_ emissions in the port areas as the average efficiency of ship engines is significantly lower than that of power plants. The marine diesel engine has large fuel consumption (e.g. a marine engine with a power output of 10,000 kW consumes approximately 5000 g/kWh of fuel) and the power generated from the engine accounts for only 30÷45% of the total fuel energy with the remainder being discharged as waste heat (Zhi-Min et al. 2019). While the efficiency of the Italian electric network is equal to 46% as with Resolution EEN 3/08, dated 20/03/2008, the Regulatory Authority for Energy Networks and the Environment (ARERA) establishes that the conversion factor of electricity into primary energy is equal to 0.187×10^−3^ toe/kWh. Furthermore, the use of significantly better filtration and abatement technologies and much more efficient environmental controls, compared to those of the ships circulating around the world today, provides environmental benefit to this technology. Cold ironing also allows reducing the acoustic impact in the port areas (Santander et al. 2018; Schiavoni et al. 2022). Powering ships moored in the port docks from land is generally complex and requires high technological and expensive infrastructures. This complexity grows further in the European ports, due to the fact that electricity is mostly distributed for over 200 meter ships (cruise, container and cargo ships) at 60 Hz (American standard), while the standard European frequency of the electricity grid is at 50 Hz. This makes it necessary to convert the frequency from 50 to 60 Hz (Espinosa et al. 2016). Although these critical issues, the Italian government is firmly intentioned to invest in these interventions and properly decided to provide a 700 M€ to the electrification of the platforms (Cold ironing) in the National Recovery and Resilience Plan (Italian government 2021), to minimize the dependence on fossil fuels and the environmental impact in the sector of maritime transport. The share of the NRRP investment provided for AdSP was equal to 50 M€ to realize the action “Stretto Verde-Sustainable maritime mobility in the area between Reggio Calabria and Messina,” funded by the Minister of Ecologic Transition. The aim of the action is to enhance the energy transition of maritime mobility in the area of the Strait of Messina, including the ports of Messina, Milazzo, Villa San Giovanni and Reggio Calabria. The benefits to introduce a sustainable maritime mobility in the Strait of Messina deal with the reduction of polluting emissions, caused by the transport of goods and people, the comply with one of the European objectives “Recharge and Refuel flagship area” and the equipping ports with infrastructures that can reduce CO_2_ emissions. In coherence with the time schedule of this Ministerial action (Fig. 5), AdSP provided to the assignment of technical-economic feasibility design services on January 2023. Later, it awarded, at the end of March 2023, the open tender procedure regarding the joint assignment of the final and executive design and the execution of the electrification works of the docks of the Port System Authority of the Strait. The contract was awarded to a temporary consortium of companies for an amount of approximately 15 M€. INVITALIA, the National Agency for Inward Investment and Economic Development, owned by the Italian Ministry of Economy, was chosen as the central purchasing body, in charge of to provide and publish the call for tender.

**Fig. 6:**
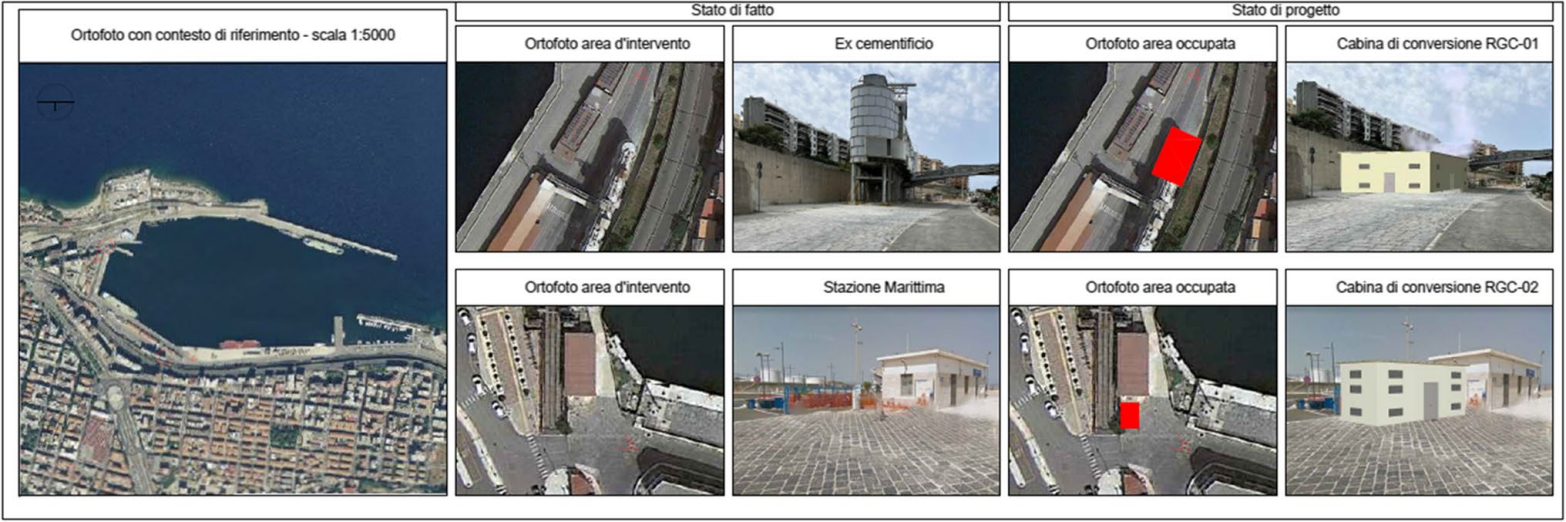
3D views of the actual state and the project of No. 2 cabins in Reggio Calabria (source: technical procurement documents published by INVITALIA)

Apart from the technical feasibility, in terms of power, energy consumption and electric network structure, the study on which the contract provide the assessment of costs and general economic convenience of the investment, in terms of depreciation and fairness of the selling prices of the service to the ship-owners. In the port of Messina, the most powerful infrastructure was projected. It is a system with 4 cabins, including those supplying the railway system. The port of Messina was designed to allow the mooring of both routine vessel traffic (railway vessels, ferries, hydrofoils, tugs, etc.) and cruise ships. The study considers the need of 20kV for the docks and transform it on site to 400V, including winches for the cable dispensers. The overall power for the port of Messina will be around 22 MVA. In the port of Milazzo, the main ships that it is intended to serve with cold ironing are the tugboats, being preponderant. The system is made of No. 5 transformation substations (No. 4 of 400 kVA and No. 1 of 1250 kVA) with output for a 60Hz-440V converter from shore and the overall power will be around 4.4 MVA. The port of Reggio Calabria was designed to moor both routine ship traffic (ferries, hydrofoils, etc.) and cruise ships. It is a system with No. 3 cabins of various types (Fig. 6), and the overall power will be equal to 19 MVA (AdSP 2022).

#### Potential conversion of ships to new fuels as LNG and hydrogen

**Fig. 7:**
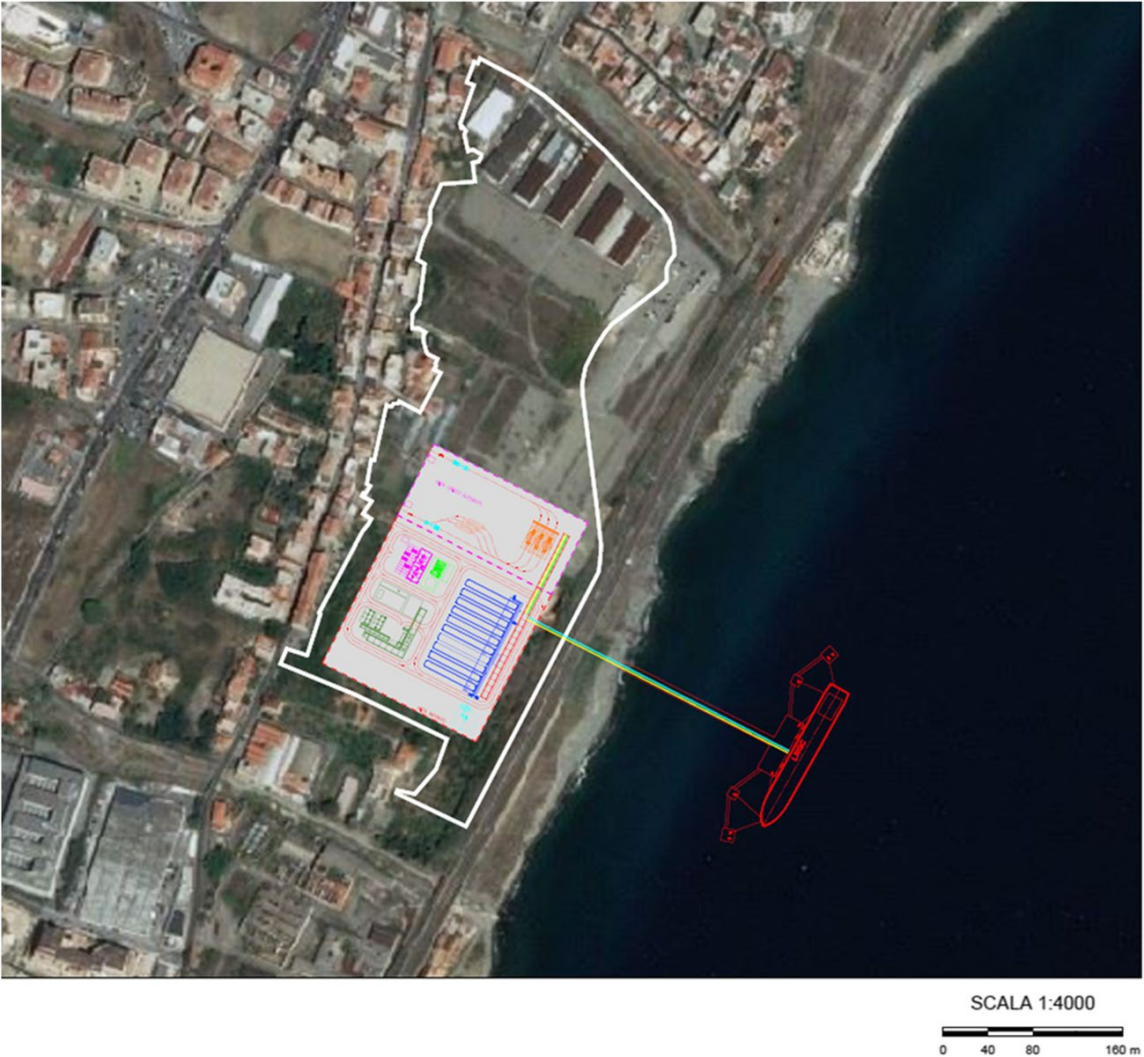
The preliminary layout of the SSLNG in the San Filippo area (Nappi 2022)

The replacement of fossil fuels with LNG is a well-known way to achieve low-carbon ship transport, particularly in lower emissions of particles, NOx and CO_2_ (Bengtsson et al. 2012; Burel et al. 2013; Smith 2010). Even if emissions of total hydrocarbons and CO are lower for LNG compared to present marine fuel oils, therefore it is not possible not to consider the methane slip from combustion of LNG (Anderson et al. 2015). Ren and Lützen (2017) developed a multi-criteria decision-making, according to which nuclear power has been recognized as the most sustainable alternative energy source for shipping, followed by LNG and wind power. Considering the results of the nuclear power referendum of November 1987, after the Chernobyl disaster, the Italian government decided to phase out the existing nuclear power plants in 1988. The following referendum of June 2011 confirmed the banning of the nuclear power; thus, this 1st option is at the moment not attainable and Italian policy towards clean energy transition is, among other measures, oriented to the use of alternative energy sources, particularly photovoltaic and wind power plants (Servizio studi Senato 2011). The interest towards LNG is still increasing in Italy. At the moment several projects are being evaluated and authorized by the competent authorities, consisting of small scale coastal storages (SSLNG) for LNG offloading from small size carriers, storage and subsequent loading onto ship-borne lighters (bunkering), and cryogenic tankers, for the refueling of civil and industrial customers and fuel filling stations, settled in Sardinia, Ravenna and Porto Marghera, and Naples (MASE 2019). Even AdSP, aiming at implementing the LNG storage activity in the south of Italy, has commissioned a feasibility study to RINA Consulting Ltd (Nappi 2022). The study provides the construction of a 9000 m^3^ SSLNG, equipped with infrastructures for bunkering and loading tankers, to be settled in a 110,000 m^2^ empty area well-known as San Filippo, 2 km north of the port of Tremestieri (Fig. 7). The aim of the SSLNG, supplied by gas tankers, has to be the loading of the barges, in the way they can supply the ships moored in the ports of the Strait and to carry out the loading of tankers that can allow the distribution of LNG by road. The main characteristics of the SSLNG are as follows:

- Maximum LNG discharge rate was from the LNG tanker of 1500 m^3^/h.
- LNG and Boil-Off Gas (BOG) return arms must be adequately sized to fit valve locations collectors of LNG carriers.
- BOG will be sent to the vessel via a header and steam return arm.
- The mooring layout will be designed considering the mooring of 30,000 m^3^ gas carriers and ships bunker with LNG storage capacity from 3000 up to 7500 m^3^.

LNG can serve as bridging technology since it does not offer a route to full decarbonization, that is a prerogative of hydrogen. Thanks to the absence of GHG, green hydrogen and hydrogen-based fuels are ideal candidates as alternative fuels in the maritime sector. Hydrogen can be used in fuel cells (FC), obtaining electricity through an electrochemical reaction (and not through the combustion process) and represents the main component of the traction systems of the Fuel Cell Hybrid Electric Vehicles (FCHEV). There are several types of fuel cells, suitable for different uses (Table 6), and the most prominent for maritime transportation uses are PEM and AFC.

**Table 6:**
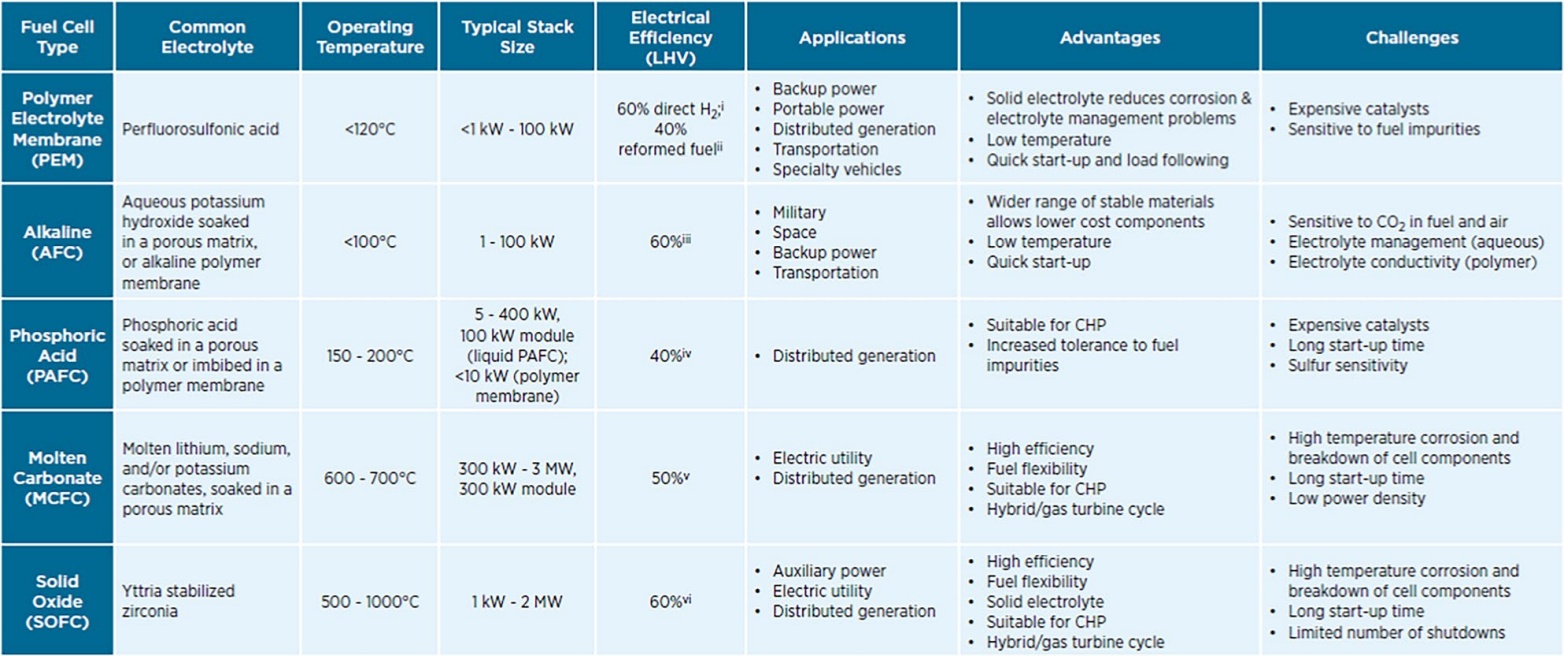
Comparison of FC technologies (US Department of Energy 2016)

**Fig. 8:**
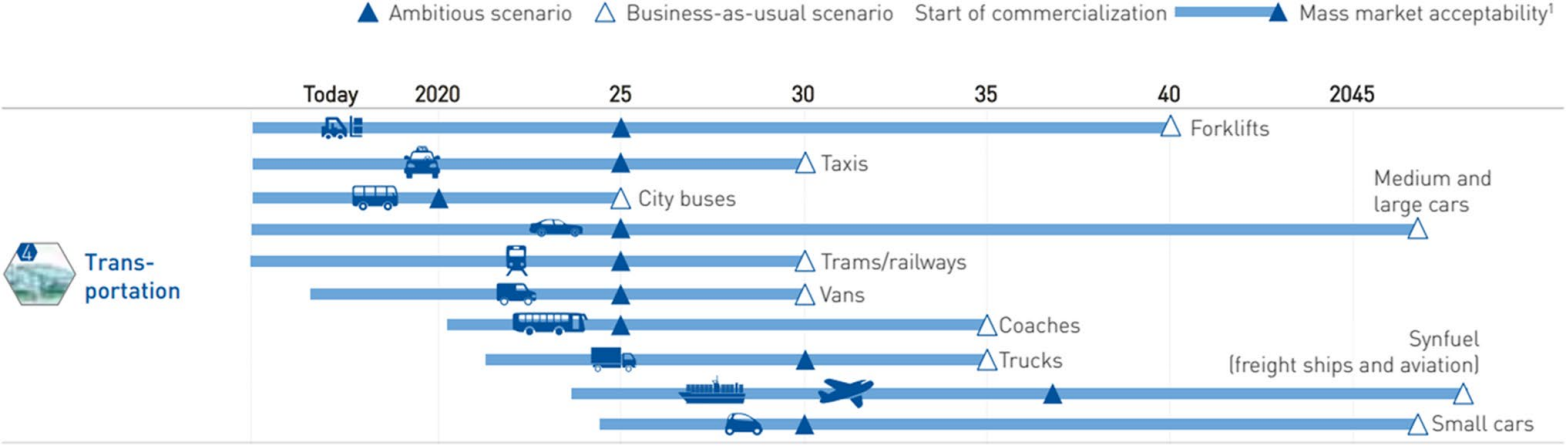
The potential for hydrogen in two scenarios (source: FCH2 Joint Undertaking 2019)

Hydrogen Europe, representing the European hydrogen and fuel cells industry, developed two scenarios, ambitious and business as usual (BAU), foreseeing the deployment of hydrogen in the transportation field (Fuel Cells and Hydrogen 2 Joint Undertaking 2019). Figure 8 shows the potential for hydrogen in the two scenarios. The ambitious scenario considers the need for a joint effort by investors, industries, and policymakers and an increase of activities, as hydrogen could play a fundamental role in decarbonization. Instead, the BAU scenario foresees a significantly lower potential for hydrogen, if higher cooperation and investments do not take place. Freight ships and aviation should reach mass-market acceptability, intended as sales up to 1% within the segment, of hydrogen and synthetic fuels derived from hydrogen, around 2038 in the ambitious scenario or 2050 in the BAU scenario. As synthetic fuel, hydrogen could replace about 4% of the EU’s fuel supply for airplanes and freighters.

**Fig. 9:**
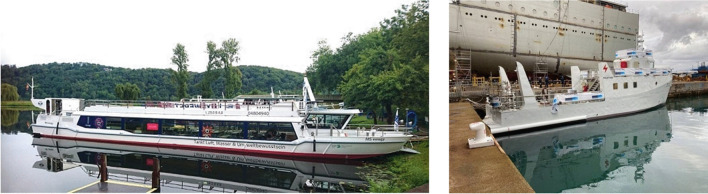
On the left, the MS Innogy (Maritime Executive 2017); on the right, The Zeus (Hydrogen-news.it 2022)

For water transport, fuel cells are most suitable for larger passenger ships, requiring longer autonomy. The environmental benefits for passengers consist of lower local emissions, less noise, and less water pollution. Besides propulsion, fuel cells can provide auxiliary power on ships, replacing diesel-based units. Prototypes for fuel-cell-powered passenger ships are already in operation, including the MS Innogy, endowed with a methanol fuel cell system, in Germany, and the Zeus, powered by 50 kg liquid hydrogen and FC based on metal-hydride technology, in Italy (Fig. 9).

**Fig. 10:**
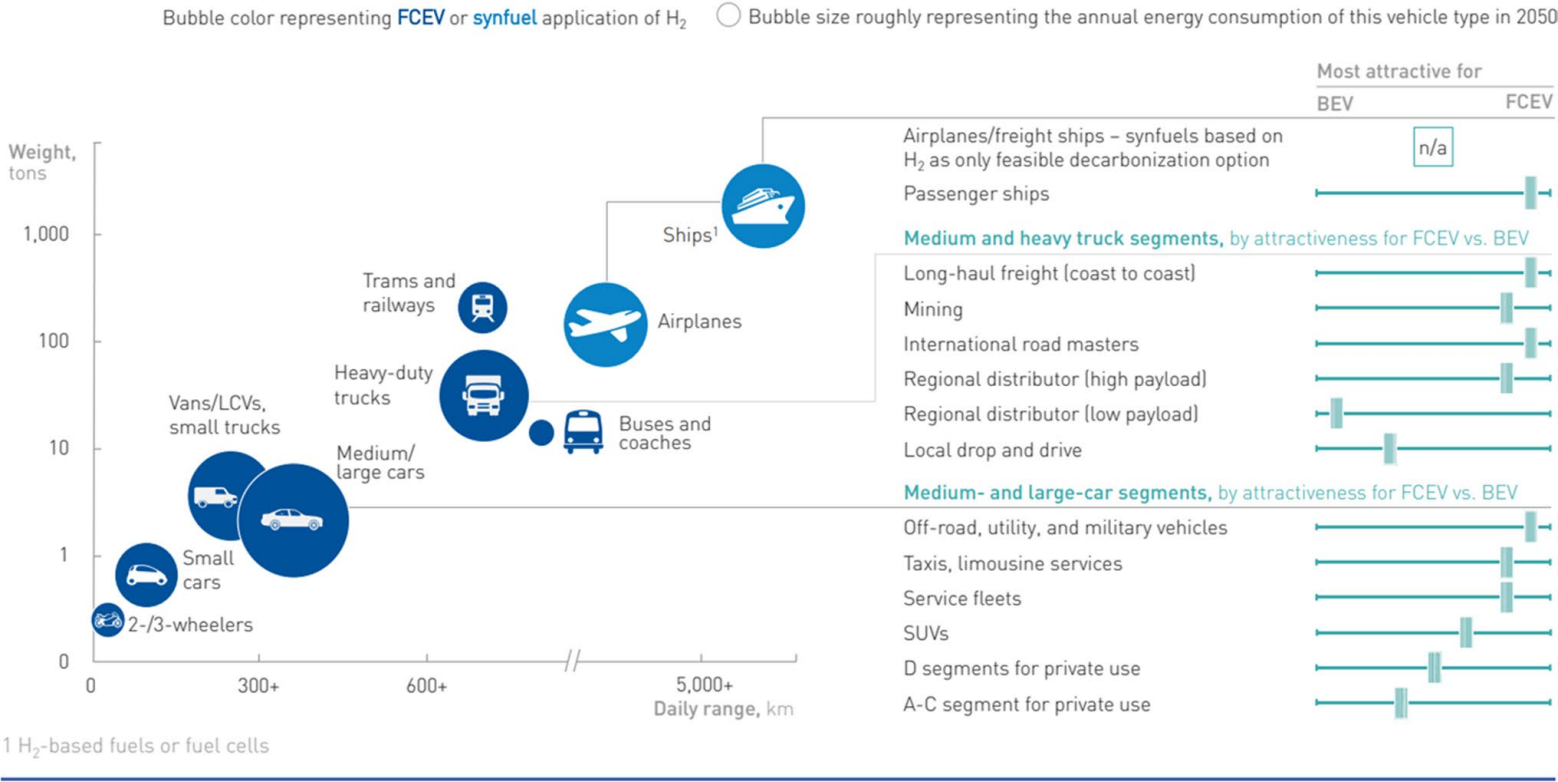
Comparison of range, payload and preferred technology (source: FCH2 Joint Undertaking 2019)

Hydrogen can also be converted into synthetic fuel by adding CO_2_ from the atmosphere. The main advantage of synfuels is to achieve the energy density of current fuels and to be chemically very similar to existing fuels. This means existing infrastructures and, in some cases engines, can be directly used with synthetic fuels, facilitating the potential use. The biggest challenge for synfuels is their lower conversion efficiency, which means higher amounts of hydrogen production for the same amount of final energy. Figure 10 shows the most suitable technologies for the various means of transport. Hydrogen-based synthetic fuels represent the main feasible decarbonization option for airplanes and freight ships.

Besides, the use of hydrogen in the maritime sector has to deal with the main technical-economic barriers that have hindered its large-scale adoption. Mainly, the costs and the low volumetric density, making the storage of hydrogen compared to other fuels difficult and secondly the absence of a specific regulatory framework, both in terms of technical regulation and policy.

#### Energy production from photovoltaic plants

**Fig. 11:**
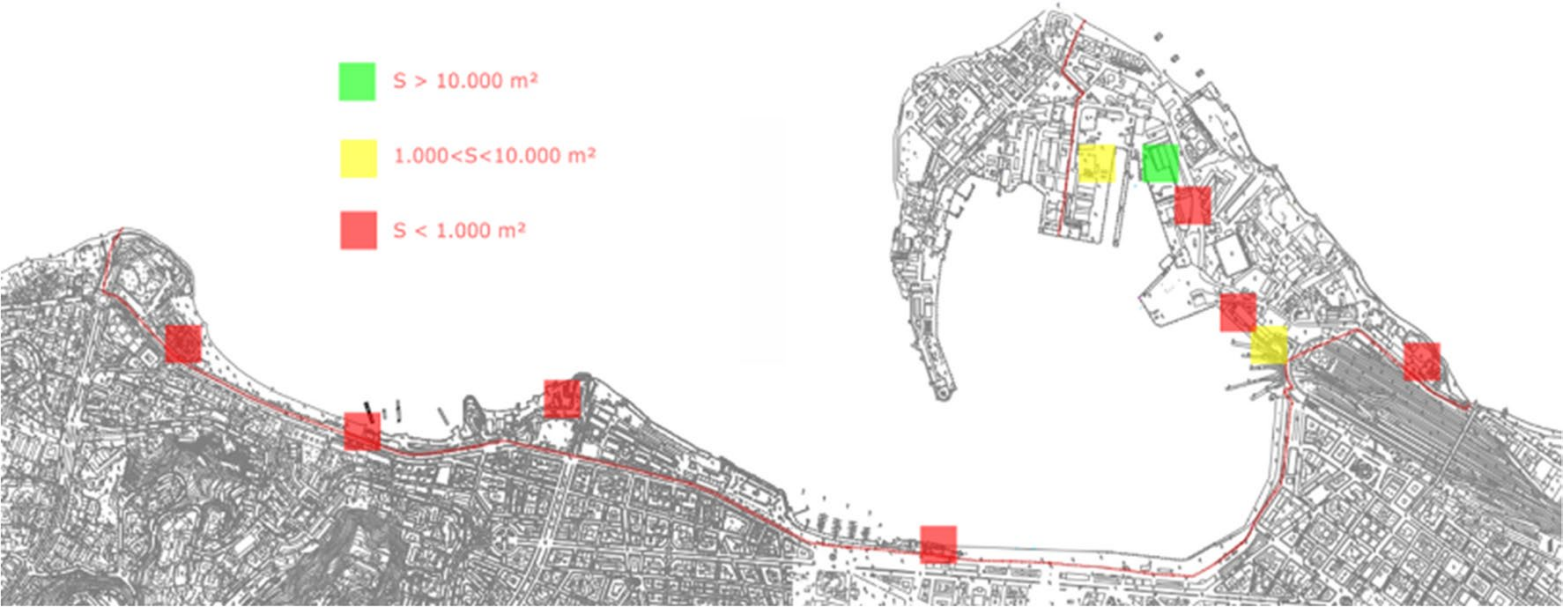
The potential sites to realize PV plants in the port of Messina

On-site survey was carried out in the port of Messina, the most suitable for morphology of the built context, to assess the areas potentially covered by photovoltaic plants, on warehouses and buildings’ coverings. The results of the study show more than 36,000 m^2^ could be suitable (Fig. 11), with Pick Power of more than 5400 kW and the production of more than 81,000 MWh/y electricity, covering from 13.5 to 60% the consumptions of the concessionaires operating inside the port of Messina.

#### Energy production from tidal currents

**Fig. 12:**
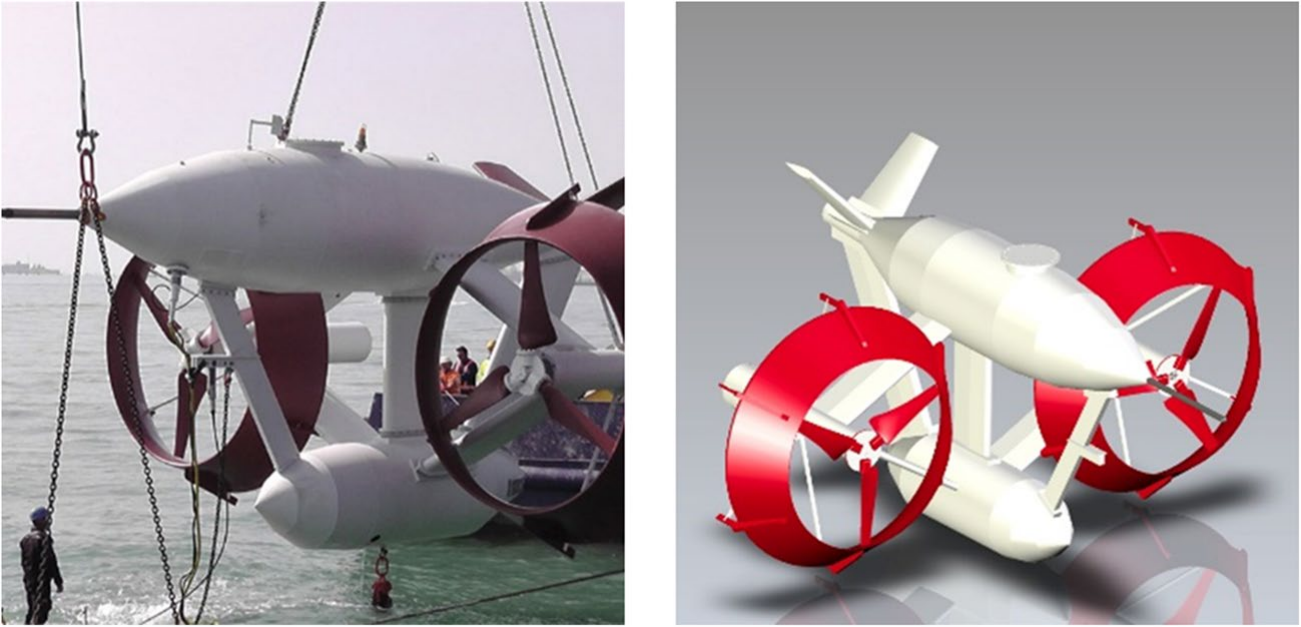
Images and rendering of the GEMSTAR® (source: https://www.gemstar.it/)

The Intergovernmental Panel on Climate Change (IPCC) recognized ocean energy as a means of mitigating climate change (IPCC 2019). Ocean energy technologies are commonly set in two main fields: tidal stream and wave energy converters, on the base of the resource utilized to generate energy. The potential of ocean energy technologies amounts to 45,000 TWh, by combining more than a dozen sources, thus ocean energy could cover more than twice the current global demand for electricity (IRENA 2020). The choice of the tidal currents to produce renewable energy arises from the peculiarity of the Strait of Messina, which is the main Italian site for the intensity of tidal currents. In Italy, the areas significantly involved by tidal currents are the Strait of Messina and the Lagoon of Venice, with high difference in speed between the two areas: in the Strait of Messina it reaches up to values equal to 3 m/s (6 knots), in the Venetian Lagoon, however, up to 1.5 m/s (3 knots). The energy potential from tidal resources in the Strait of Messina was calculated in 125 GWh/y, by exploiting only the areas near the coasts, both Calabrian and Sicilian ones, without considering the central part of the Strait, being the same affected by naval traffic (Coiro et al. 2013). Among others, the DEASP presents one of the more promising technologies: the GEMSTAR® by Seapower. It belongs to the family of submerged floating plants and can represent an excellent opportunity, thanks to features capable of reducing installation and maintenance costs, with a lower levelised cost of electricity (LCOE) of 0.11 USD/kWh, comparable with other systems developed up to now in Worldwide, as the current LCOE for tidal is estimated at between USD 0.20/kWh and USD 0.45/kWh (IRENA 2020). The GEMSTAR® (Fig. 12) consists of two horizontal axis hydrokinetic turbines, mounted on the sides of a floating, submerged structure, able to align with the flow even during tidal inversion.

The structure consists of a streamlined hollow body provided with a self-towing winch, allowing easier emersion and immersion maneuvers. Each rotor is coupled to an electric generator case in each of the side nacelles. An inverter can be used for the connection to the electrical grid and the power connection is provided by means of a power cable, laid out along the mooring cable and extended up to an on-shore grid connection point. The system is held in position, at the desired depth, by means of an anchorage system to the seabed consisting of a winch, an anchor block positioned on the seabed and a mooring cable. Unlike systems set on the seabed, it is able to optimize the exploitation of the marine currents thanks to the possibility of easily regulating its depth, simply by winding or releasing the mooring cable around the winch. (Coiro et al. 2020). A 100 kW prototype was deployed and tested in the Venice Lagoon, thus reaching TRL 8, with an expected average early production of 300 MWh with 2.5 m/s maximum speed site.

### Cost-benefit analysis

AdSP considers to realize its environmental and energy strategy by means of the aforementioned interventions, just funded in part. For each intervention, the environmental impact in terms of GHG emissions reduction was calculated and the results were reported in the following Table 7, considering also the provided period of application.

**Table 7:** The interventions toward the decarbonization of the AdSP ports

Type of intervention	Period [years]	Investment costs [M€]	GHG reduction [tCO_2equ_]
Electrification of port docks—“Cold Ironing”	20	20	13,630
Spreading of the supply of LNG ships and vehicles	15	90	4,020
Charging stations and purchase of electric vehicles	10	0.05	237
Improving the efficiency of the building envelope	10	0.18	–
Improving the efficiency of the public lighting network	10	0.5	233
Photovoltaic systems on roofs of buildings	25	8.2	3,355
Experimental studies for the production of tidal energy	20	10	1,455
**Total**		**128.93**	**22,930**

All the interventions, at the end of the next 25 years, when they will be completed, will produce a significant GHG emissions reduction, with higher contribution due to the Cold Ironing intervention. If applied at the current situation, considering separately the situation with and without the EI, these interventions will provide the following previsions reported in Table 8.

**Table 8:** AdSP carbon footprint, considering the application of the sustainable strategy at the current status [tCO_2equ_]

Source	2019 (including EI)	2019 (excluding EI)	2020 (including EI)	2020 (excluding EI)
Concessionaries	4,525,722	50,113	5,074,473	40,780
AdSP sustainable strategy	22,930	22,930	22,930	22,930
Percentage reduction [%]	0.51	45.76	0.45	56.22

The impact of these interventions on the carbon footprint of AdSP including EI is insignificant, but this is due to the peculiarities of these two industrial plants, whose sustainable strategy to reduce GHG emissions is developed by themselves. The GHG emissions reduction was calculated in more than 56%, considering the GHG emissions produced by concessionaries without EI in 2020.

## Conclusions

The sustainable energy strategy of the DEASP of the Port System Authority of the Strait was defined starting from the analysis of the current state through a characterization of the ecological footprint of the main activities taking place in the managed port areas. These assessments were therefore fundamental for a short-medium term forecast assessment in order to direct the AdSP energy-environmental choices in accordance with the most recent national and international guidelines on decarbonization. The AdSP port system showed a good potential for reduction of primary energy consumption and the associated GHG emissions thanks to the implementation of a sustainable energy strategy, based on a mix of energy transition actions ranging from conventional solutions (solar and tidal energy, energy efficiency of public lighting and buildings, electric vehicles, etc.) to more innovative solutions (cold ironing and the transition to LNG and at long term to hydrogen). With an investment plan of more than 128 M€, the DEASP estimated the reduction of almost 23,000 tCO_2equ_ in the next 20 years, thus reducing to more than half the GHG emissions in the port context. Due to this deepen analysis, it was possible to learn some lessons:

1. High difference in ports’ consumptions, not necessarily depending on its surface

- 1000 MWh/y Reggio Calabria
- 33,000 MWh/y Villa San Giovanni
- 125,000 MWh/y Messina and Tremestieri (cruise traffic)
- 17,000,000 MWh/y Milazzo (refinery and power station)

2. High difference in CO_2_ emissions
3. Different cost-benefit estimation for several interventions focused on the clean energy transition of the port system

The Italian Government, in the last 2 decades, pointed as strategic infrastructure on the Messina Bridge (Italian government 2021). On 31/03/2023, the President of the Republic has issued the decree law containing “Urgent provisions for the construction of the stable connection between Sicily and Calabria” and authorized the relative bill to be presented to the Chambers. With Law 26/05/2023 No. 58 the timing for the approval of the executive project was fixed to 31/07/2024. This infrastructure will certainly contribute to the reduction of environmental pollution, due to the reduction of the goods trade by ships, which will be turned to the bridge. Additional information on the environmental impact of the Messina Bridge will be available with the conclusion of the project phase.

## Data Availability

All the data and materials presented in the paper were in part acquired by the AdSP concessionaries or directly by AdSP and elaborated by the authors.
